# Evidence for novel polycyclic aromatic hydrocarbon degradation pathways in culturable marine isolates

**DOI:** 10.1128/spectrum.03409-23

**Published:** 2023-12-12

**Authors:** Jillian L. Walton, Alison Buchan

**Affiliations:** 1 Department of Microbiology, University of Tennessee, Knoxville, Tennessee, USA; Connecticut Agricultural Experiment Station, New Haven, Connecticut, USA

**Keywords:** co-metabolism, marine bacteria, *Roseobacteraceae*, PAH degradation, bioremediation

## Abstract

Polycyclic aromatic hydrocarbons (PAHs) are common toxic and carcinogenic pollutants in marine ecosystems. Despite their prevalence in these habitats, relatively little is known about the natural microflora and biochemical pathways that contribute to their degradation. Approaches to investigate marine microbial PAH degraders often heavily rely on genetic biomarkers, which requires prior knowledge of specific degradative enzymes and genes encoding them. As such, these biomarker-reliant approaches cannot efficiently identify novel degradation pathways or degraders. Here, we screen 18 marine bacterial strains representing the Pseudomonadota, Bacillota, and Bacteroidota phyla for degradation of two model PAHs, pyrene (high molecular weight) and phenanthrene (low molecular weight). Using a qualitative PAH plate screening assay, we determined that 16 of 18 strains show some ability to degrade either or both compounds. Degradative ability was subsequently confirmed with a quantitative high-performance liquid chromatography approach, where an additional strain showed some degradation in liquid culture. Several members of the prominent marine *Roseobacteraceae* family degraded pyrene and phenanthrene with varying efficiency (1.2%–29.6% and 5.2%–52.2%, respectively) over 26 days. Described PAH genetic biomarkers were absent in all PAH degrading strains for which genome sequences are available, suggesting that these strains harbor novel transformation pathways. These results demonstrate the utility of culture-based approaches in expanding the knowledge landscape concerning PAH degradation in marine systems.

**IMPORTANCE:**

Polycyclic aromatic hydrocarbon (PAH) pollution is widespread throughout marine environments and significantly affects native flora and fauna. Investigating microbes responsible for degrading PAHs in these environments provides a greater understanding of natural attenuation in these systems. In addition, the use of culture-based approaches to inform bioinformatic and omics-based approaches is useful in identifying novel mechanisms of PAH degradation that elude genetic biomarker-based investigations. Furthermore, culture-based approaches allow for the study of PAH co-metabolism, which increasingly appears to be a prominent mechanism for PAH degradation in marine microbes.

## INTRODUCTION

Polycyclic aromatic hydrocarbons (PAHs) are pollutants generated from incomplete combustion of organic compounds, such as wood, coal, petroleum oil, and municipal solid waste, that are ubiquitously found in atmospheric, terrestrial, and aquatic environments (1, 2). PAHs are composed of fused aromatic rings and are classified based on their molecular weight: low (<4 rings; LMW) and high (>4 rings; HMW). These compounds are toxic and carcinogenic, presenting a hazard to both human and environmental health (2). The removal of these compounds from the environment is difficult due to their stability, hydrophobicity, and low bioavailability (2). Due to their recalcitrance and hazardous nature, the U.S. Environmental Protection Agency has designated 16 PAHs as priority pollutants (3). HMW PAHs are of particular concern as the recalcitrance of these chemicals increases with the number of aromatic rings (4). While chemical and physical methods have been designed to remove PAHs from the environment, many of these methods have low efficiency and high costs relative to biological removal methods, including microbial degradation (4).

Marine ecosystems are frequently the final destinations for PAHs through terrestrial run-off, atmospheric deposition, industrial discharge, and oil spills (2, 5, 6). Despite the abundance of PAHs in marine ecosystems, relatively few marine bacteria have been investigated for PAH biodegradation pathways. In contrast, significant research has been conducted with soil and freshwater PAH degrading bacteria, including *Mycobacterium* spp., *Pseudomonas* spp., and *Sphingomonas* spp. (7). While, in principle, these bacteria can be applied to marine environments for bioaugmentation, such approaches have challenges, most notably bacterial survival and the ability to degrade a compound in non-native environments (8). Additionally, halophilic and halotolerant marine bacteria may be useful in remediation of high-salinity waste effluents that commonly contain PAH contaminants, such as industrial wastewater (9, 10). To effectively remediate marine environments and high-salinity wastes, marine bacteria isolated from ecosystems of interest should be investigated for their ability to degrade PAHs.

Bacteria are capable of transforming PAHs in various ways. The marine sediment bacterium *Mycobacterium vanbaalenii* PYR-1 was one of the first strains demonstrated to utilize pyrene, a HMW PAH, as a sole carbon source. The enzymes required for the complete mineralization of several PAHs have been characterized in this strain (11 – 13). Most other characterized PAH degraders employ similar enzymatic reactions to those identified in *M. vanbaalenii* PYR-1. Accordingly, the *M. vanbaalenii* PYR-1 pathway and highly homologous pathways form the basis for identification of PAH degradation capabilities in both isolated strains and in culture-independent gene surveys. In general, complete PAH catabolism is initiated by hydroxylation of an aromatic ring (via a dioxygenase or monooxygenase), followed by subsequent re-aromatization and ring cleavage reactions, resulting in monocyclic intermediates that are funneled to central metabolism (i.e., tricarboxylic acid cycle). Not all PAH degraders are capable of complete mineralization, instead, transforming these compounds into less toxic intermediates through hydroxylation and addition of methoxy groups (12). Metabolically, these intermediates are dead-end products, and further degradation is generally prevented due to the methylation of hydroxyl groups (1). Finally, co-metabolism of PAHs is increasingly observed in diverse bacteria. This phenomenon usually occurs with LMW and HMW PAH mixtures, where the presence of more labile LMW PAH increases the rate or efficiency of HMW PAH degradation (4, 14, 15). While some evidence exists that other labile carbon sources (e.g., starch and yeast extract) can be used for co-metabolism of PAHs, less information is available about the mechanism of co-metabolism and which compounds effectively induce co-metabolism (16, 17).

Several genetic biomarkers for PAH degradation have been established. First, is the initial ring-hydroxylating dioxygenase (RHD), which catalyzes the initial rate-limiting step for PAH degradation: hydroxylation of an aromatic ring. These enzymes (e.g., PahA and NidA) have been reported to be substrate specific but may be able to hydroxylate several different ring structures (13, 18). Specifically, the alpha subunit of this enzyme is responsible for substrate specificity and is used as a biomarker for PAH degradation. Second, the PAH hydratase-aldolase (*pahE*) has been shown to be the most specific and conserved biomarker within conventional PAH degradation pathways. This is the first step from which an organism may gain energy from the breakdown of PAHs through liberation of substrates feeding into the TCA cycle (19). Third, the final ring-cleaving reaction acts on the monocyclic aromatic derivatives to produce TCA cycle intermediates, supporting growth. Generally, PAHs are converted into monoaromatic central intermediates, protocatechuate or catechol, and funneled through their respective catabolic pathways (20). Each of these three steps provides a necessary reaction for complete PAH mineralization in conventional degradation pathways.

A variety of culture-independent approaches are used to probe for PAH degraders in marine environments, including PCR amplification of biomarker genes (most often PAH RHD), 16S rRNA gene libraries of PAH enrichments, and biomarker homology and identity searches in metagenomes and genomes (21 – 23). These approaches have broadened the view of PAH degradation in marine habitats by increasing the number of predicted PAH degraders, identifying phylogenetic distribution of genetic biomarkers, and discovering diverse genetic organization of pathways (19, 24, 25). For example, a recent study demonstrated the utility and specificity of *pahE* as a genetic biomarker and used an amplicon-based library to recover sequences that led to the prediction of PAH degradative abilities for several previously unreported taxa (e.g., *Nevskia ramosa* DSM 11499 and *Rhodovulum* sp. N122) (19). While these culture-independent approaches have increased our knowledge of the genetic diversity and distribution of putative PAH degraders, these efforts frequently fail to: (i) link functionality to specific organisms and (ii) identify novel pathways for PAH degradation. Additionally, the focus on only those isolates capable of using PAHs (single or mixed) as a sole carbon source limits our understanding of strains that may play critical roles in co-metabolic transformation of PAHs in the environment.

To aid in expanding the landscape of known marine PAH degraders and pathways, we screened 18 diverse marine bacterial isolates for the ability to degrade pyrene and/or phenanthrene. After our initial screen, we focused our efforts on members of the marine *Roseobacteraceae* family as prior studies have indicated that representatives (e.g., *Roseovarius* species, *Ruegeria pomeroyi* DSS-3, and *Celeribacter indicus* P73) of this abundant and active group of bacteria are able to degrade both LMW and HMW PAHs, including pyrene, phenanthrene, and fluoranthene (17, 24, 26). From this work, we hypothesize that some marine bacteria: (i) harbor novel pathways for PAH degradation and (ii) may likely not degrade PAHs as a sole carbon source, explaining the limited marine bacterial PAH degraders and pathways identified via omics-based approaches and culture-based approaches that investigate sole metabolism of PAHs.

## RESULTS

### PAH degradative abilities identified in diverse marine bacteria

An initial panel of 12 marine bacteria representing three phyla (Pseudomonadota, Bacillota, and Bacteroidota) abundant in marine ecosystems was subjected to a qualitative PAH degradation assay using pyrene and phenanthrene top agar plates (*Marinobacterium georgiense* DSM 11526, *Bacillus-Clostridium* strain SE165, *Bacillus-Clostridium* strain SE98, *Alteromonas macleodii* EZ55, *Vibrio natriegens* ATCC 14048, *Rhodospirillaceae* strain EZ35, *Flavobacteriaceae* strain EZ40, *Alcanivorax* sp. strain EZ46, *Ruegeria pomeroyi* DSS-3, *Citreicella* sp. SE45, and *Sagittula stellata* E-37). *R. pomeroyi* DSS-3 has previously been shown to degrade PAHs, thus this screening confirmed its degradative ability (17). Except for *Alcanivorax* sp. strain EZ46 and *M. georgiense* DSM 11526*,* all tested strains showed clearing zones, indicative of degradation, on both pyrene and/or phenanthrene top agar plates containing complex medium after 7 days (Table 1). In contrast, no convincing indication of degradation was evident for strains on the PAH top agar plates containing minimal medium, suggesting that these strains could not utilize PAHs as sole carbon sources (Table 1; Fig. S1). *Citreicella* sp. SE45 did show clearing zones on the PAH top agar plates with minimal medium; however, this was attributed to the strain’s ability to use the acetone solvent as a carbon source (data not shown).

**TABLE 1:** Screening of marine strains using pyrene and phenanthrene top agar plate assay

Bacterial strain	Taxonomic phyla/class	Complex + pyrene	Complex + phenanthrene	Minimal + pyrene	Minimal + phenanthrene
*Marinobacterium georgiense* DSM 11526	Gammaproteobacteria	−^*b*^	−	−	−
*Bacillus-Clostridium* strain SE165	Firmicutes	+^*a*^	+	−	−
*Bacillus-Clostridium* strain SE98	Firmicutes	+	+	−	−
*Alteromonas macleodii* EZ55	Gammaproteobacteria	+	+	−	−
*Vibrio natriegens* ATCC 14048	Gammaproteobacteria	+	+	−	−
*Rhodospirillaceae* strain EZ35	Alphaproteobacteria	+	+	−	−
*Flavobacteriaceae* strain EZ40	Bacteroidetes	+	+/−^*c*^	−	−
*Alcanivorax* sp. strain EZ46	Gammaproteobacteria	−	−	−	−
*Ruegeria pomeroyi* DSS-3	Alphaproteobacteria	+	+	−	−
*Citreicella* sp. SE45	Alphaproteobacteria	+	+	+	+
*Sagittula stellata* E-37	Alphaproteobacteria	+	+	−	−
*Escherichia coli* DH5α	Gammaproteobacteria	−	−	−	−

^*a*^ "+” denotes clearing zones evident by day 7 or 14.

^*b*^ "−” denotes no clearing zones evident by day 7 or 14.

^*c*^ “+/−” denotes inconclusive clearing zone.

Following the initial screen, efforts were focused on members of the marine *Roseobacteraceae* family, an abundant and active group of heterotrophic bacteria with known abilities to degrade lignin-derived aromatic compounds (27) (Fig. S2). Three PAH degradation positive strains from the initial screen are family members (*R. pomeroyi* DSS-3, *Citreicella* sp. SE45, and *S. stellata* E-37). Several additional *Roseobacteraceae* were subsequently screened (*Sulfitobacter* sp. EE-36, *Sulfitobacter* sp. NAS-14.1, *Ruegeria* sp. TM1040, *Roseovarius* sp. 217, *Roseovarius nubinhibens* ISM, Rhodobacterales strain Y4I, and *Sulfitobacter pontiacus* CB-D) using the PAH top agar plate assay with complex medium (Table 2). All showed clearing zones on the pyrene-containing complex medium plates, and all but one (*R. nubinhibens* ISM) showed definitive clearing on phenanthrene-containing complex medium plates. Rhodobacterales strain Y4I produces a blue pigment that stains the agar, obfuscating any clearing. To assess the PAH degradation of this organism using the PAH top agar assay, the assay was repeated with an unpigmented variant (*igiD*::Tn5) (28). This strain showed clearing zones on both pyrene and phenanthrene top agar plates with complex medium (Fig. S3).

**TABLE 2:** Screening of *Roseobacteraceae* strains using pyrene and phenanthrene top agar plate assay

*Roseobacteraceae* strains	Complex + pyrene	Complex + phenanthrene
*Ruegeria* sp. TM1040	+^*a*^	+
*Ruegeria pomeroyi* DSS-3	+	+
*Sulfitobacter* sp. EE-36	+	+
*Sulfitobacter* sp. NAS-14.1	+	+
*Sulfitobacter* sp. CB-D	+	+
*Roseovarius nubinhibens* ISM	+	+/−^*c*^
*Roseovarius* sp. 217	+	+
Rhodobacterales strain Y4I (*igiD*::Tn5)	+	+
*Citreicella* sp. SE45	+	+
*Sagittula stellata* E-37	+	+
*Escherichia coli* DH5α	−^*b*^	−

^*a*^ "+” denotes clearing zones evident by day 7.

^*b*^ "−” denotes no clearing zone evident by day 7.

^*c*^ “+/−” denotes inconclusive clearing zone.

### Quantitative assessment of PAH degradative ability

**Fig 1:**
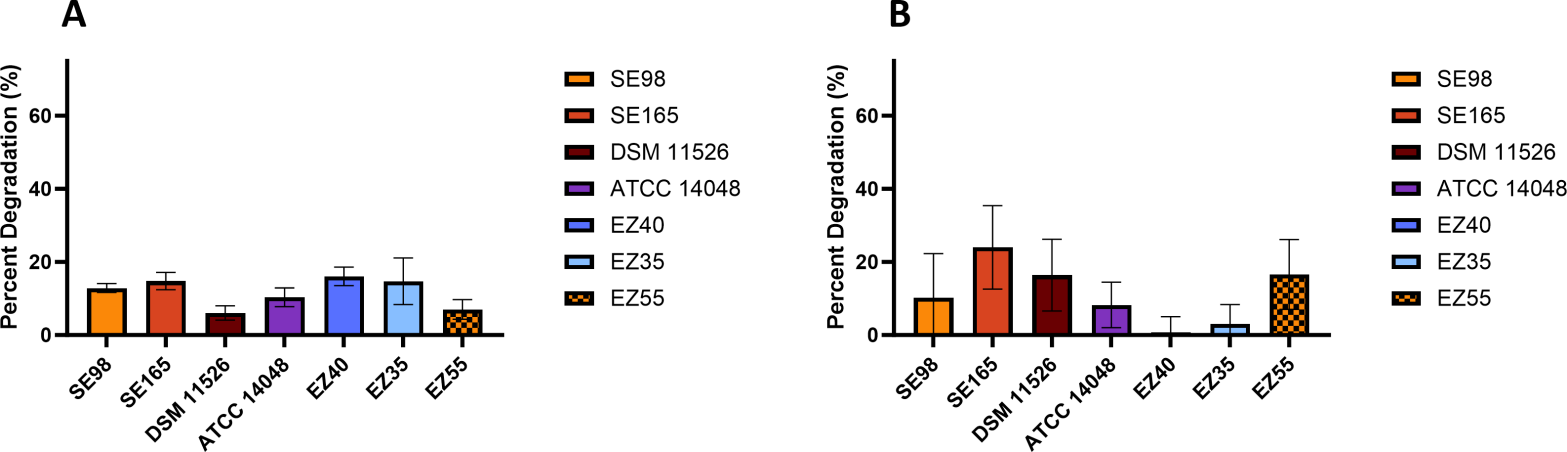
Degradation of (**A**) pyrene and (**B**) phenanthrene by marine strains after 26 days. Percent degradation is relative to T0 culture and accounts for deviation from uninoculated controls. Standard error was calculated from three replicate cultures. Strain designations are indicated on *x*-axis.

For all strains, excluding *Alcanivorax* sp. EZ46, PAH loss was quantified using HPLC (Fig. 1). All results were consistent with the PAH top agar assay, except *M. georgiense* DSM 11526, which, despite not showing clearing zones on the PAH top agar plates, showed 6% and 16.4% degradation of pyrene and phenanthrene, respectively, in liquid culture. The extent of pyrene degradation for the marine strains ranged from 6% to 16.1% with *Rhodospirillaceae* strain EZ35, *Bacillus-Clostridium* strain SE165, and *Flavobacteriaceae* strain EZ40 showing the highest average pyrene degradation at 14.7%, 14.8%, and 16.1% loss, respectively. Phenanthrene degradation ranged from 0.7% to 24% with *M. georgiense* DSM 11526, *Alteromonas macleodii* EZ55, and *Bacillus-Clostridium* strain SE165 having the highest average phenanthrene degradation at 16.4%, 16.6%, and 24% loss, respectively. While *Flavobacteriaceae* strain EZ40 showed the greatest degradation of pyrene, it exhibited the lowest for phenanthrene in agreement with the PAH top agar assay. Only three strains, *Bacillus-Clostridium* strain SE165, *M. georgiense* DSM 11526, and *Alteromonas macleodii* EZ55, showed greater degradation of phenanthrene relative to pyrene.

**Fig 2:**
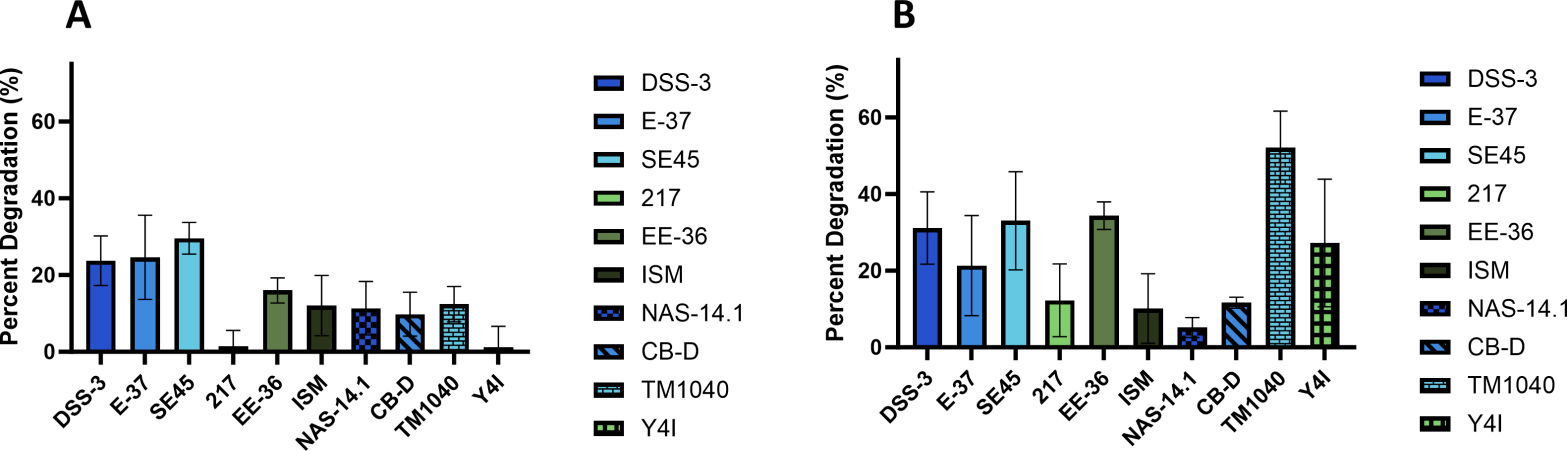
Degradation of (**A**) pyrene and (**B**) phenanthrene by *Roseobacteraceae* strains after 26 days of incubation. Percent degradation is relative to T0 culture and accounts for deviation from uninoculated controls. Standard error was calculated from three replicate cultures. Strain designations are indicated on *x*-axis.

For *Roseobacteraceae* strains, pyrene degradation ranged from 1.2% to 29.6% (Fig. 2). *R. pomeroyi* DSS-3, *S. stellata* E-37, and *Citreicella* sp. SE45 had the highest pyrene degradation at 23.8%, 24.6%, and 29.6%, respectively. Most of the *Roseobacteraceae* strains showed greater degradation of phenanthrene degradation relative to pyrene ranging from 5.2% to 52.2%. Notably, *Ruegeria* sp. TM1040 showed the greatest difference from 12.4% loss of pyrene to 52.2% loss of phenanthrene. *Citreicella* sp. SE45, *Sulfitobacter* sp. EE-36, and *Ruegeria* sp. TM1040 had the highest phenanthrene degradation averaging 33.1%, 34.4%, and 52.2%, respectively. The *Roseobacteraceae* strains also appeared to have a greater range of degradative abilities for both pyrene and phenanthrene relative to other marine strains.

### PAH degradation protein identity in marine strains

**Fig 3:**
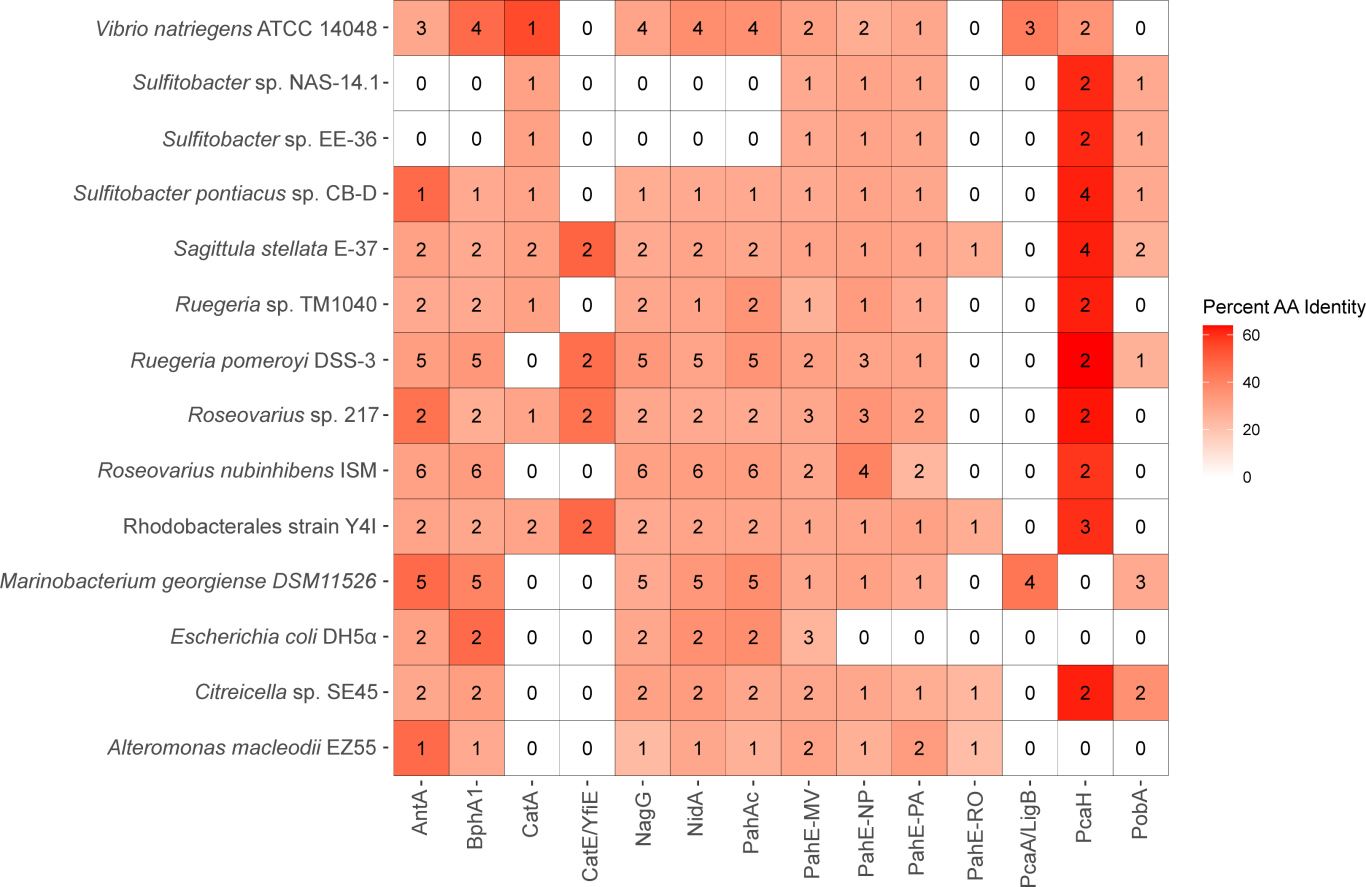
Summary of PAH protein BLASTP protein identity searches. Boxes are color-coded based on the highest percent identity of the protein hits from each strain. The numbers in the boxes indicate protein hits for each query sequence below an E-value of 1E^−10^. White boxes with a “0” indicate no hits with an E-value below 1E^−10^. Protein query sequences are named either as the protein abbreviation or as the protein abbreviation with the first letter of the genus and species from which the query sequence originated, as indicated in the text. Detailed results from the protein identity searches are in Table S2.

An analysis of putative PAH degradation pathways and genes was conducted for strains with available genome sequences: *M. georgiense* DSM 11526, *A. macleodii* EZ55, *V. natriegens* ATCC 14048, *R. pomeroyi* DSS-3, *Citreicella* sp. SE45, *S. stellata* E-37, *Sulfitobacter* sp. EE-36, *Sulfitobacter* sp. NAS-14.1, *Sulfitobacter* sp. CB-D, *Roseovarius* sp. 217, *Ruegeria* sp. TM1040, *Roseovarius nubinhibens* ISM, and Rhodobacterales strain Y4I. *E. coli* DH5α, a non-PAH degrader was included for reference (Fig. 3). The analysis was focused on conserved reactions common to most characterized PAH degradation pathways: (i) initial PAH ring-hydroxylation via RHD; (ii) TCA substrate liberation via PAH hydratase-aldolase; (iii) monocyclic aromatic hydrocarbon catabolism via RHD (11, 12, 29). Protein sequence alignment searches were conducted with representative protein sequences using BLASTP and results returning an E-value below 1E^−10^ were further considered (Tables S2 and S3; Fig. 3). NidA (Gram + PAH/Phthalate RHD), PobA (Group I RHD), AntA (Group II RHD), PahAc (Group III RHD), BphA1 (Group IV RHD), and NagG (Salicylate RHD) were chosen to represent the known diversity of aromatic ring-hydroxylating dioxygenases involved in the first conserved reaction (30). PahE protein sequences from *Novosphingobium pentaromativorans* US6-1 (PahE-NP), *Rhodococcus opacus* B4 (PahE-RO), *Mycobacterium vanbaalenii* PYR-1 (PahE-MV), and *Pseudomonas aeruginosa* PaK1 (PahE-PA) were chosen as representatives of PAH hydratase-aldolase diversity (the second conserved reaction). Four different monocyclic aromatic dioxygenases were chosen, two from pathways that degrade protocatechuate (PcaH and PcaA/LigB) and two from pathways that degrade catechol (CatA and CatE/YfiE).

All strains appeared to be missing proteins involved in PAH-specific degradation (Fig. 3). Most strains encode putative protein sequences with an E-value below 1E^−10^ for non-PAH specific RHD groups, with identities ranging from 22% to 36% for Group I (PobA), 22% to 47% for Group II (AntA), and 24% to 47% for Group IV (BphA1). The Salicylate Group RHD, NagX, had no strong protein hits. For NidA (Gram + PAH/Phthalate RHD) and PahAc (Group III RHD), *E. coli* DH5α had a higher identity than almost all protein hits for both of these query sequences, with 36% (E-value 1E^−91^) and 37% (E-value 2E^−88^) identity, respectively. The two exceptions are *V. natriegens* ATCC 14048 with 37% identity (E-value 1E^−88^) to NidA and *M. georgiense* DSM 11526 with 37% (E-value 1E^−88^) identity for PahAc (Table S3). None of the strains appear to encode a putative PahE protein, most showing <30% sequence identity. For all strains, PahE-MV identities were ≤31% (E-value ≤5E^−27^), PahE-NP identities were ≤40% (E-value ≤2E^−22^), PahE-PA identities were ≤33% (E-value ≤1E^−15^), and PahE-RO identities were ≤27% (E-value ≤3E^−12^). Interestingly, only *R. nubinhibens* ISM had any PahE identity greater than 35%, at 40% for PahE (E-value 5E^−19^). Most of the putative PahE results were annotated as dihydropicolinate synthases or related enzymes, which are commonly found in microorganisms where they are expected to play a role in lysine biosynthesis (31). The PcaA/LigB, PcaH, CatE/YfiE, and CatA proteins are involved in the lower pathway of PAH degradation, steps that occur after monocyclic aromatic compounds are formed. *Roseobacteraceae* strains encode similar proteins to PcaH (≤59% identity) (32). Other than the *Roseobacteraceae* strains, only *V. natriegens* ATCC 14048 encoded proteins with similarity to PcaH (35%; E-value ≥1E^−18^). No *Roseobacteraceae* strains had any similar proteins for PcaA/LigB, whereas *V. natriegens* ATCC 14048, *E. coli* DH5α, and *M. georgiense* DSM 11526 all showed proteins with >30% identity to PcaA/LigB. Thus, of the strains with genomes analyzed, only *A. macleodii* EZ55 appears to be missing enzymes involved in protocatechuate degradation. Of the catechol dioxygenases, Rhodobacterales strain Y4I, *Roseovarius* sp. 217, and *S. stellata* E-37 had protein hits for both investigated dioxygenases (CatA and CatE/YfiE) with ≤31% and ≤49% identities, respectively. Out of the non-*Roseobacteraceae* strains, only *V. natriegens* ATCC 14048 had a protein hit for CatA. While no strains showed >40% identity to proteins specific to upper PAH degradation pathway proteins (i.e., PahE, NidA, and PahAc), proteins involved in lower PAH degradation pathways strains had much higher protein identities.

### *Roseobacteraceae* PahE biomarker homology

**Fig 4:**
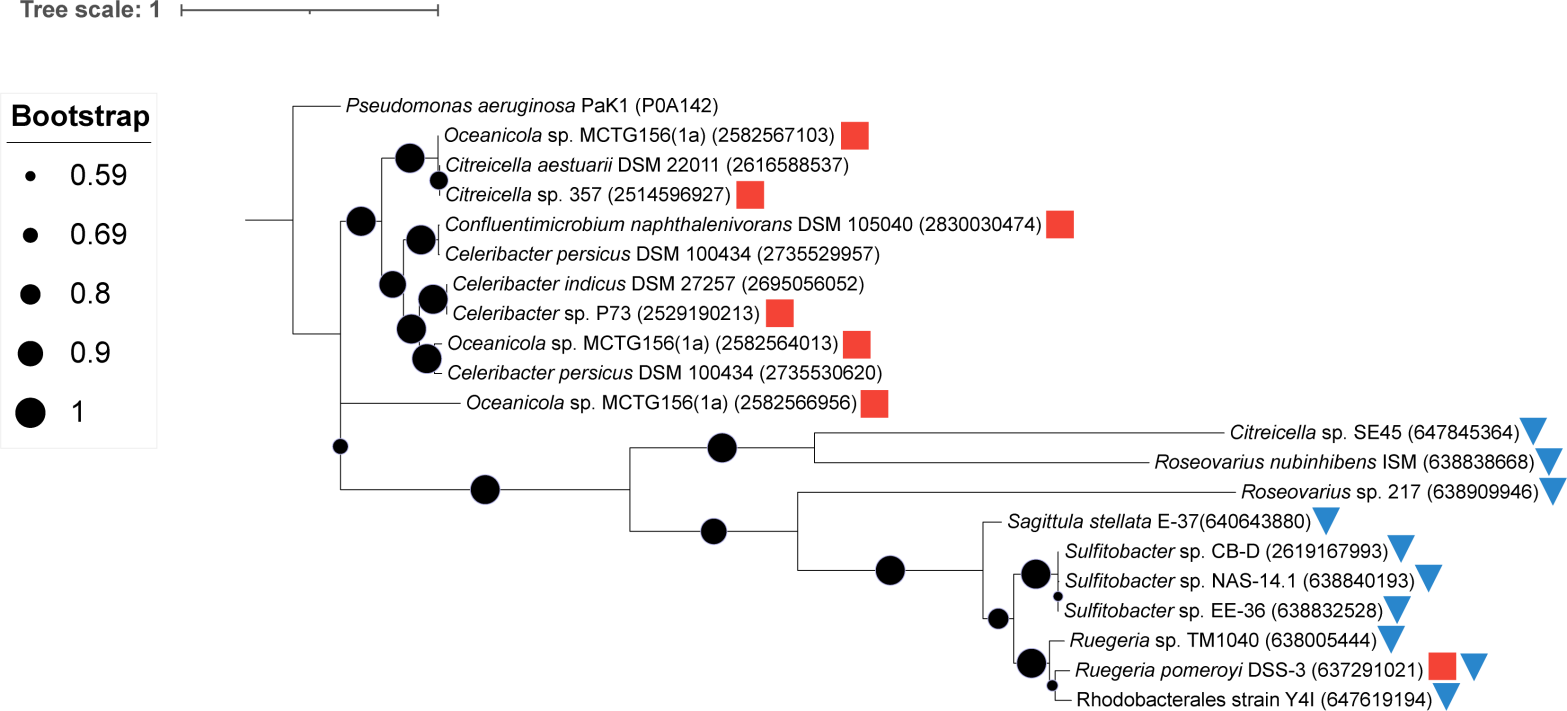
Maximum likelihood phylogenetic tree of select *Roseobacteraceae* to PahE homologs. Red squares indicate prior reports of PAH degradation for a given strain (17, 19, 26, 35). Blue triangles indicate strains for which PAH degradation ability was demonstrated in this study. Protein accession numbers are provided in parentheses. Bootstrap values (1,000 iterations) are shown at branch nodes with circle size corresponding to the value as indicated in the key. The scale bar represents the number of substitutions per site. *P. aeruginosa* PaK1 was used and included as the original query sequence that identified the putative PahE proteins.

To further explore the *Roseobacteraceae* family and their potential PAH degradation ability, over 750 *Roseobacteraceae* genomes were searched for PahE proteins. Only seven genomes had >50% identity (E-value ≤2E^−133^) to the PahE from *Pseudomonas aeruginosa* PaK1 (UniProt ACN P0A142) (Fig. 4). Of the *Roseobacteraceae* collection analyzed, all possessed at least one similar protein but at low identity (≤31%). Clear phylogenetic differences appear between these two groups of bacteria. *Roseobacteraceae* strains with over 50% identity to PahE had several bacteria previously reported to degrade PAHs (Fig. 4) (26, 33 – 36). Additionally, putative PahE homologs of *Roseobacteraceae* strains screened in this study were investigated for the presence of conserved residues that would support the activity of these enzymes in PAH degradation. This analysis revealed that the putative PahE homologs are missing several active site residues although some strains possess the catalytic residue (Fig. S4).

## DISCUSSION

To expand the knowledge of bacterial PAH degradation in marine systems, 18 strains representing diverse bacterial taxa were screened for their ability to transform phenanthrene (LMW PAH) and pyrene (HMW PAH). From an initial collection of 12 strains, three *Roseobacteraceae* strains demonstrated the highest degradation of PAHs (~20% degradation by 26 days relative to <20% for the remaining taxa) in a quantitative assay. As such, additional family members were included in subsequent quantitative analyses. Despite the fact that none of the strains assayed here were derived from PAH enrichments nor from contaminated sites, all but one (*Alcanivorax* sp. strain EZ46) showed evidence of pyrene and/or phenanthrene transformation was under the cultivation conditions used. *Roseobacteraceae* family members are well known for their ability to degrade lignin-derived monocyclic aromatic compounds, and recent evidence suggests that some strains can transform PAHs (17, 21, 22, 35). For example, *R. pomeroyi* DSS-3 has been previously reported to degrade phenanthrene, pyrene, and benzo[a]pyrene in complex media containing additional carbon sources (tryptone and yeast extract) (17). Similarly, *Flavobacteriaceae* and marine *Alteromonas* strains have been strongly implicated in PAH as well as oil degradation due to the appearance of strains in enrichment cultures (5, 37, 38). Consistent with prior reports of numerous *Bacillus* species, the two *Bacillus-Clostridium* strains examined in this study were able to degrade pyrene and phenanthrene (39, 40). In contrast, reports of PAH degradation by other Gammaproteobacterial genera (*Vibrio*, *Rhodospirillaceae*, and *Marinobacterium*) included in the collection screened here are scant. A *Vibrio cyclotrophicus* strain has been shown to degrade LMW PAHs but not use them as a growth substrate (41). *Rhodospirillaceae* strains have been identified in PAH enrichment experiments, but little specific information exists regarding their ability to transform PAHs (42, 43). *Marinobacterium georgiense* isolate (IAM 1419T) was found in a PAH-enriched microbial consortium, but no confirmation of its degradation has been reported (42). Finally, *Alcanivorax* species are known degraders of n-alkanes from oil hydrocarbons, but little evidence exists regarding their ability to degrade PAHs, aligning with the findings of this study (44, 45). Marine bacteria are recognized to possess high metabolic and physiological diversity among closely related strains, thus variation in PAH degradation abilities is unsurprising (46 – 48). However, it does highlight the necessity of documenting PAH degradation ability of marine isolates, even within closely related strains, if we are to improve our understanding of the ecology and evolution of these degraders.

While none of the strains were able to utilize PAHs as a sole carbon source, nearly all strains showed PAH degradation when also provided labile carbon substrates in the complex medium (yeast extract and tryptone), suggestive of co-metabolism of pyrene and phenanthrene among this cohort of marine bacteria. Co-metabolism, the synergistic degradation of two carbon sources, is a common feature among PAH degraders, with most studies reporting the synergistic breakdown of HMW PAHs and LMW PAHs (49, 50). Co-metabolism of PAHs with non-aromatic, labile carbon sources is recognized, but often not considered in PAH degradation studies (16, 51). This study emphasizes the need to assess both sole-metabolism and co-metabolism of PAHs to identify contributing degraders in natural environments. In addition to the carbon source provided, growth mode appears to also influence degradative abilities in some strains. For example, *M. georgiense* DSM 11526 showed degradation only in liquid culture and not with PAH overlay plates. Exploring co-metabolic growth substrates as well as growth conditions will provide further evidence for the role of marine microbes in natural attenuation of PAHs.

While a plethora of strains have been found to degrade LMW PAHs, fewer degraders have been discovered that transform HMW PAHs, presumably due to their decreased bioavailability and increased stability. All but one strain analyzed, here, showed some ability to degrade both a HMW PAH and a LMW PAH, with many strains demonstrating greater degradation of LMW PAHs. While these compounds are generally degraded by substrate-specific RHDs, flexibility in these enzymes is evident with some acting on both LMW and HMW PAHs (13, 18). The available genomes analyzed revealed that strains encoded genes with low identity to PAH-specific RHDs, suggesting that non-PAH-specific RHDs act on these compounds. This is consistent with evidence that some marine bacteria use various RHDs, with broad substrate ranges, to degrade PAHs (26, 52). We suggest enzymes involved in the degradation of other aromatic compounds may act on PAHs, as has been demonstrated for the *Roseobacteraceae* member, *Celeribacter indicus* P73 (26). All *Roseobacteraceae* members possessed proteins with high protein identity to PcaH, a marker for protocatechuate degradation (32). Additionally, of the remaining strains, both *M. georgiense* and *V. natriegens* are predicted to encode enzymes that may be required for lower pathways of PAH degradation. It is important to recognize, however, that enzymes involved in the lower pathway of PAH degradation lack the specificity to solely be used as PAH degradation biomarkers due to the plethora of compounds that are funneled through these pathways (53). It is also relevant to highlight that high protein identity to specific RHDs does not necessarily indicate an ability to degrade PAHs. For example, *E. coli* DH5α had higher protein identities to NidA and PahAc than most strains tested in this study, yet this strain showed no evidence of degradation in the assays. Collectively, these findings indicate that strains may use different enzymes than those present in conventional PAH degradation pathways. Finally, further studies are needed to assess whether any strains utilize PAH-derived carbon for biosynthetic or energetic purposes.

PahE has been recently implicated as a biomarker due to its specificity for PAH degradation and its conservation across taxonomically diverse organisms but could not reliably indicate PAH degradation ability with strains in this study (19, 50, 54). To consider the utility of this gene as an indicator for PAH degradation, a broader analysis of 750 *Roseobacteraceae* genomes showed low sequence identity and an absence of conserved residues to validate PahE proteins. This approach was also unable to identify many known *Roseobacteraceae* PAH degraders from this study and others (17, 24). Due to the lack of PahE, it is possible that these strains have novel pathways for the biodegradation of PAHs that are not currently detected by bioinformatic approaches that rely on such biomarkers as indications of PAH degradation ability.

PAHs are common pollutants in marine ecosystems, and degradation of these pollutants frequently occurs via native bacteria, albeit at limited rates (6, 37). Consequently, PAH degraders are crucial to systems subject to contamination and serve as potential candidates for remediation purposes and indicators of active PAH biodegradation. Bioinformatic and omics-based research depends on prior culture-based work to define genetic biomarkers for PAH degradation. Current biomarkers have challenges with specificity for PAH degradation pathways and the ability to identify novel pathways and/or PAH degraders. The evidence presented by this study suggests that we have yet to uncover the full diversity of bacterial PAH degraders as well as biochemical pathways employed to transform these compounds. Bridging the gap between culture-based investigations and modern bioinformatic approaches holds the key to elucidating the full landscape of PAH biodegradation in marine ecosystems.

## MATERIALS AND METHODS

### Bacterial strains

The following 18 strains were analyzed in this study: *Marinobacterium georgiense* DSM 11526 (55), *Bacillus-Clostridium* strain SE165 (56), *Bacillus-Clostridium* strain SE98 (56), *Alteromonas macleodii* EZ55 (57), *Vibrio natriegens* ATCC 14048 (44), *Rhodospirillaceae* strain EZ35 (57), *Flavobacteriaceae* strain EZ40 (57), *Alcanivorax* sp. strain EZ46 (57), *Ruegeria pomeroyi* DSS-3 (45), *Citreicella* sp. SE45 (56), *Sagittula stellata* E-37 (58), *Sulfitobacter* sp. EE-36 (59), *Sulfitobacter* NAS-14.1(60), *Ruegeria* sp. TM1040 (48), *Roseovarius* sp. 217 (61), *Roseovarius nubinhibens* ISM (45), Rhodobacterales strain Y4I (28), *Sulfitobacter pontiacus* CB-D (62), and *Escherichia coli* DH5α (see Table S1 for strain descriptions). These strains were routinely grown on YTSS (yeast tryptone sea salt) agar [per liter: 15 g Instant Ocean (Thermo Fisher Scientific), 15 g agar (Thermo Fisher Scientific), 4 g tryptone, 2.5 g yeast extract] or YTSS broth at 30°C in the dark, unless otherwise noted.

### PAH degradation screening plates

To screen for PAH degradation, a modification of the plate screening assay described in Bogardt and Hemmingsen was used (63). For this modified screening assay, strains were inoculated on top of a PAH-containing top agar rather than within the top agar layer. This allows colonies to be scraped off the agar surface to evaluate clearing zones directly beneath the colonies. In addition, we decided to use a complex medium to screen marine strains for co-metabolism in conjunction with screening initial marine strains for degradation of PAHs as a sole growth substrate (17). For PAH co-metabolism screening, YTSS agar was used as a complex medium wbase layer, and, for PAH degradation as a sole carbon source, aromatic basal media (ABM) agar [ABM—per liter 8.7 mM KCl, 8.7 mM CaCl_2_, 43.5 mM MgSO_4_, and 174 mM NaCl with 225 μM K_2_HPO_4_, 13.35 mM NH_4_Cl, 71 mM Tris-HCl (pH 7.5), 15 g agar (Thermo Fisher Scientific), 68 μM Fe-EDTA, trace metals (7.85 mM nitrilotriacetic acid, 0.53 mM MnSO_4_·H_2_O, 0.42 mM CoCl_2_·6H_2_O, 0.35 mM ZnSO_4_·7H_2_O, 0.038 mM CuSO_4_, 0.11 mM NiCl_2_·6H_2_O, 1.16 mM Na_2_SeO_3_, 0.41 mM Na_2_MoO_4_·2H_2_O, 0.33 mM Na_2_WO_4_·2H_2_O, 0.25 mM Na_2_SiO_3_·9H_2_O) and trace vitamins [0.0020% vitamin H (Biotin), 0.0020% folic acid, 0.0100% pyridoxine-HCl (B6), 0.0050% riboflavin (B2), 0.0050% thiamine (B1), 0.0050% nicotinic acid, 0.0050% pantothenic acid (B5), 0.0001% cyanocobalamin (B12), 0.0050% *p*-aminobenzoic acid]] was used as a minimal medium base layer. The PAH top-agar overlay was constructed using 5 mg/mL stock solutions of pyrene or phenanthrene dissolved in acetone (Thermo Fisher Scientific, ≥99.5%) and 5 mL of 0.7% top agar, using either agar (Thermo Fisher Scientific) for complex or Agar Noble (Difco) for minimal media. The final concentration of pyrene (Millipore Sigma, 98%) and phenanthrene (Millipore Sigma, 98%) in the agar overlay was 286 µg/mL and 430 µg/mL, respectively. Due to the difference in solubility of phenanthrene relative to pyrene, a higher concentration of phenanthrene was added to the top agar to ensure precipitation. The top agar was mixed and poured evenly on the base layer plate. PAH top agar plates were left in a fume hood for at least 2 hours to allow residual acetone to vaporize.

**Fig 5:**
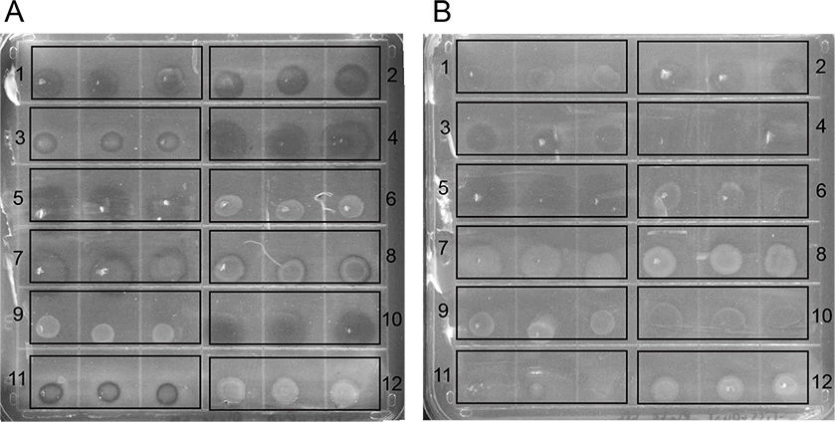
(**A**) Pyrene + complex medium and (**B**) phenanthrene + complex medium top agar assay plate after 5 days of incubation. For each strain, three replicate bacterial spots were plated as follows: (1) *R. pomeroyi* DSS-3, (2) *Citreicella* sp. SE45, (3) *S. stellata* E-37, (4) *Bacillus-Clostridium* strain SE165, (5) *Bacillus-Clostridium* strain SE98, (6) *M. georgiense* DSM 11526, (7) *V. natriegens* ATCC 14048, (8) *Rhodospirillaceae* strain EZ35, (9) *Alcanivorax* sp. strain EZ46, (10) *A. macleodii* EZ55, (11) *Flavobacteriaceae* strain EZ40, and (12) *E. coli* DH5α. Clearing zones appear as dark circles on the media and are visualized after colonies are scraped from the top agar.

To prepare strains for the PAH top agar screening assay, all strains were grown to stationary phase in YTSS broth, and densities adjusted to an optical density (540 nm) of~ 1.6. For PAH top agar complex medium plates, 5 µL of each strain was directly spotted onto plates in triplicate. For PAH top agar minimal medium plates, 1 mL aliquots was first gently centrifuged (2,200 × *g* for 5 minutes) and washed twice with ABM broth prior to inoculating plates with 5 µL of each strain in triplicate. Plates were incubated at 30°C in a polypropylene humidity chamber to prevent plates from drying out. PAH top agar complex medium plates were incubated for 7 days with two technical replicate plates scraped per day for each PAH (Fig. 5). PAH top agar minimal medium plates were incubated for 14 days with two technical replicate plates scraped after 7 days and after 14 days. One strain (*Citreicella* sp. SE45) was able to use acetone as a carbon source, complicating our ability to ascertain its ability to use PAHs as a sole carbon source. Only PAH top agar plates with complex medium were used for the *Roseobacteraceae* screen as all marine bacteria showed degradation only via co-metabolism, and previous results suggested that *Roseobacteraceae* members used in this study could not degrade PAHs as a sole carbon source (17). *E. coli* DH5α, previously reported to not degrade PAHs, was used as a negative control (19).

### HPLC quantification of pyrene and phenanthrene degradation

Marine and *Roseobacteraceae* strains (triplicates), except for *Alcanivorax* sp. EZ46, were inoculated into 10 mL 5% YTSS and grown overnight, shaking at 200 rpm. *Alcanivorax* sp. EZ46 was excluded as it would not grow in liquid culture conditions. Densities were adjusted to an OD_540_ of ~1.6 in 5% YTSS prior to inoculation (100 µL) in 9.9 mL 5% YTSS with 25 µg/mL pyrene or phenanthrene. Before inoculation, 5% YTSS and PAH tubes were incubated overnight at 30°C to evaporate off residual acetone. A subset of cultures was immediately extracted (T0 controls), and the remaining cultures were incubated for 26 days. Uninoculated controls were incubated and processed in parallel. PAHs were extracted as follows: 10 mL of HPLC-grade ethyl acetate (Thermo Fisher Scientific) was added to each tube, mixed, and allowed to separate. The top aqueous layer was transferred to a 15 mL polypropylene conical tube and directly injected into an Agilent 1100 High Performance Liquid Chromatography System (Agilent Technologies Co. Ltd). The following conditions were used with an injection volume of 20 µL: a C18 column (Acclaim PolarAdvantage II C18 5 µm 120 Å 4.6 × 250 mm, Thermo Fisher Scientific Inc.) was operated at 30°C with methanol as the mobile phase at a flow rate of 1 mL min^−1^ (17). Both pyrene and phenanthrene were detected using a UV detector at 254 nm. Peak area was compared to the initial inoculum, and peak area was normalized to uninoculated controls.

### Genomic analyses

For strain with available genome sequences: *M. georgiense* DSM 11526, *A. macleodii* EZ55, *V. natriegens* ATCC 14048, *R. pomeroyi* DSS-3, *Citreicella* sp. SE45, *S. stellata* E-37, *Sulfitobacter* sp. EE-36, *Sulfitobacter* NAS-14.1, *Ruegeria* sp. TM1040, *Roseovarius* sp. 217, *R. nubinhibens* ISM, Rhodobacterales strain Y4I, *S. pontiacus* CB-D, and *E. coli* DH5α. Protein identity searches were done using BLASTP against available genomes at the Joint Genome Institute Integrated Microbial Genomes & Microbiomes System (https://img.jgi.doe.gov/). Query amino acid sequences were selected to cover a diverse range of proteins involved in PAH degradation and obtained from NCBI. Query sequences had their function experimentally proven (Table S2) except for PahE sequences from *Novosphingobium pentaromativorans* US6-1 and *Rhodococcus opacus* B4, included to cover the known diversity of PahE proteins as previously described in Liang et al. (54).

Phylogenetic analysis was conducted for putative PahE proteins from over 750 *Roseobacteraceae* strains using BLASTP in JGI IMG (Table S4). PahE from *Pseudomonas aeruginosa* PaK1 was used as the search query. Strains that showed at least one protein over 50% amino acid identity and highest amino acid identity results for *Roseobacteraceae* strains in this study were used to construct a maximum likelihood phylogenetic tree. Protein sequences were aligned using BioEditv7.2.5 (64), and the tree was constructed using MEGAXv10.2.2 using the Jones-Taylor-Thornton evolutionary model (65). To further investigate the functionality of these proteins, we aligned putative PahE sequences of *Roseobacteraceae* strains listed in this paper with the PahE sequence from *P. aeruginosa* PaK1 (UniProt ACN P0A142). Catalytic residues and putative active sites were predicted based on conserved residues from the NCBI Conserved Domains Database (https://www.ncbi.nlm.nih.gov/cdd) of the CHBPH_aldolase subfamily, containing trans-o-hydroxybenzylidenepyruvate hydratase-aldolase and trans-2′-carboxybenzalpyruvate hydratase-aldolase, both of which are PAH hydratase aldolases.

## Data Availability

The authors confirm that all data supporting these findings are available in this article and associated supplemental materials. Raw data are available upon request to the corresponding author.

## SUPPLEMENTAL MATERIAL

Supplemental figures spectrum.03409-23-s0001.pdf Figures S1 to S4.

Table S1 spectrum.03409-23-s0002.pdf Marine strains used in this study.

Table S2 spectrum.03409-23-s0003.pdf Query sequences for protein identity searches.

Table S3 spectrum.03409-23-s0004.xlsx PAH biomarker amino acid identity search results.

Table S4 spectrum.03409-23-s0005.xlsx PahE BLASTP results for *Roseobacteraceae* family members.

ASM does not own the copyrights to Supplemental Material that may be linked to, or accessed through, an article. The authors have granted ASM a non-exclusive, world-wide license to publish the Supplemental Material files. Please contact the corresponding author directly for reuse.
