# Erratum for Walton and Buchan, “Evidence for novel polycyclic aromatic hydrocarbon degradation pathways in culturable marine isolates”

**DOI:** 10.1128/spectrum.00118-24

**Published:** 2024-02-02

**Authors:** Jillian L. Walton, Alison Buchan

## ERRATUM

Volume 12, no. 1, e03409-23, 2023, https://doi.org/10.1128/spectrum.03409-23. In the second paragraph of Acknowledgments, “OCE-1357242” should read “OCE-1737237.”

