# Supplementary material for: Evidence for novel polycyclic aromatic hydrocarbon degradation pathways in culturable marine isolates
Source: Microbiol Spectr. 2023 Dec 12;12(1):e03409-23. doi: 10.1128/spectrum.03409-23 (PMC10783047; doi:10.1128/spectrum.03409-23)
Supplement: Supplemental figures — Figures S1 to S4. [file spectrum.03409-23-s0001.pdf]

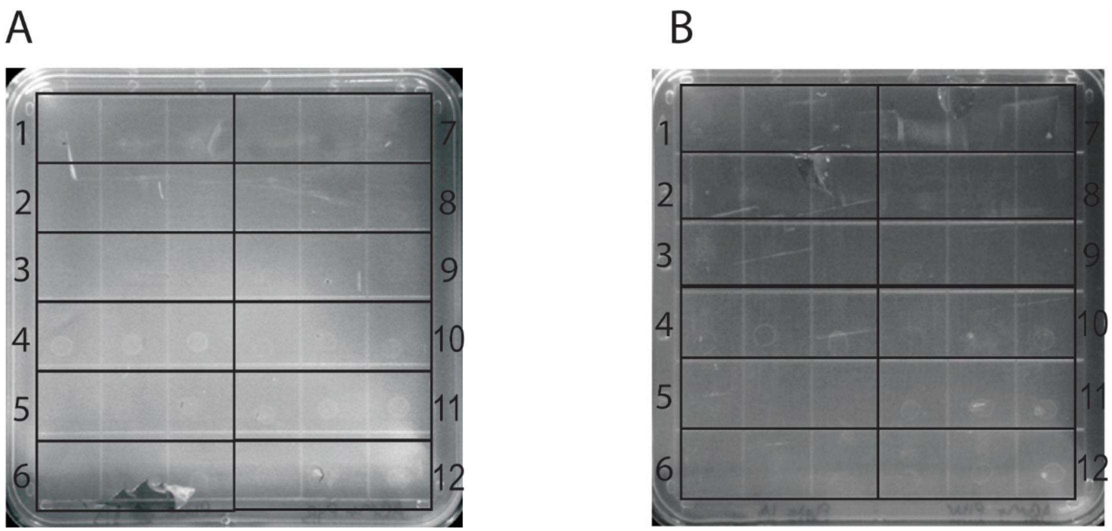

Figure S1: Minimal medium PAH plate screening for marine strains. (A) Pyrene top agar screening plates after 7 days of incubation. Colonies have been scraped from plates to score for clearing zones. B) Phenanthrene top agar screening plates after 7 days of incubation. Colonies have been scraped from plates to score for clearing zones. 1) *R. pomeroyi* DSS-3, 2) *Citreicella* sp. SE45, 3) *S. stellata* E-37, 4) *Bacillus-Clostridium* strain SE165, 5) *Bacillus-Clostridium* strain SE98, 6) *M. georgiense* DSM 11526, 7) *V. natriegens* ATCC 14048, 8) *Rhodospirillaceae* strain EZ35, 9) *Alcanivorax* sp. strain EZ46, 10) *A. macleodii* strain EZ55, 11) *Flavobacteriaceae* strain EZ40, and 12) *E. coli* DH5α. Clearing zones appear as dark circles on the media.

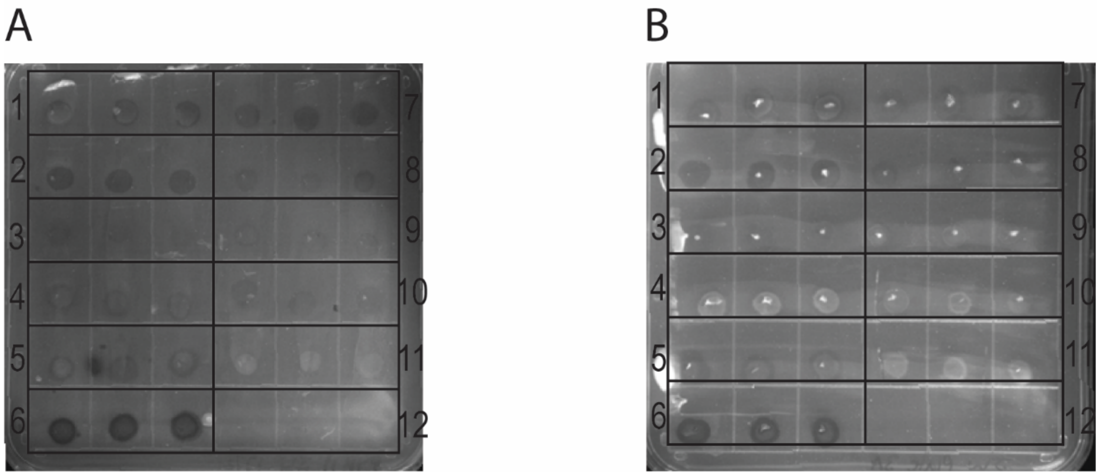

Figure S2: Complex medium PAH plate screening for *Roseobacteraceae* strains. A) *Roseobacteraceae* strains in pyrene top agar screening plates after 4 days of incubation. Colonies have been scraped from plates to score for clearing zones. B) *Roseobacteraceae* strains on phenanthrene top agar screening plates after 3 days of incubation. Colonies have been scraped from plates to score for clearing zones. 1) *R. pomeroyi* DSS-3, 2) *S. stellata* E-37, 3) *R. nubinhibens* ISM, 4) *Sulfitobacter* sp. EE-36, 5) *Citreicella* sp. SE45, 6) Rhodobacterales strain Y4I, 7) *Ruegeria* sp. TM1040, 8) *Roseovarius* sp. 217, 9) *Sulfitobacter* sp. NAS-14.1, 10) *S. pontiacus* CB-D, 11) *E. coli* DH5α, and 12) None. Clearing zones appear as dark circles on the media.

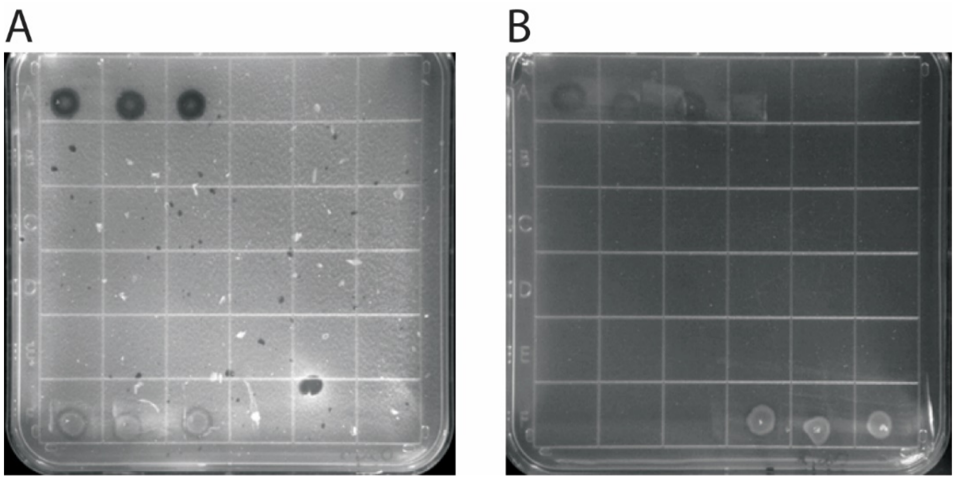

Figure S3: Rhodobacterales strain Y4I and *igiD*$^-$ pyrene and phenanthrene screening. Wild type Y4I is shown on the top of the plate and the *igiD*$^-$ mutant strain is shown on the bottom of the plate. A) Pyrene top agar screening plate and B) Phenanthrene top agar screening plate after 5 days of incubation.

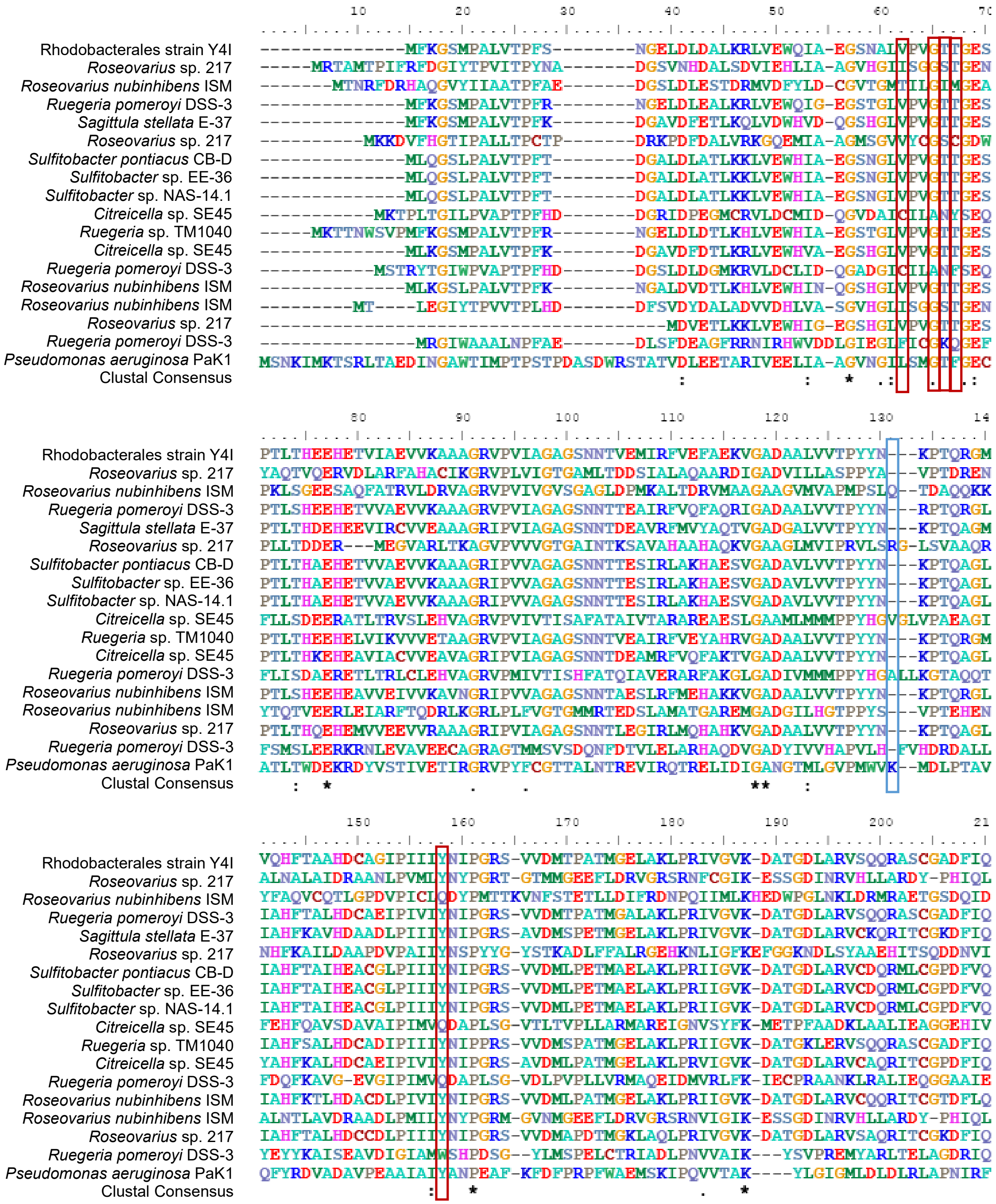
Rhodobacterales strain Y4I
Roseovarius sp. 217
Roseovarius nubinhibens ISM
Ruegeria pomeroyi DSS-3
Sagittula stellata E-37
Roseovarius sp. 217
Sulfitobacter pontiacus CB-D
Sulfitobacter sp. EE-36
Sulfitobacter sp. NAS-14.1
Citreicella sp. SE45
Ruegeria sp. TM1040
Citreicella sp. SE45
Ruegeria pomeroyi DSS-3
Roseovarius nubinhibens ISM
Roseovarius nubinhibens ISM
Roseovarius sp. 217
Ruegeria pomeroyi DSS-3
Pseudomonas aeruginosa PaK1
Clustal Consensus

Residue; : indicates Strong Similarity; · Weak Similarity). Active and catalytic sites were derived from the CHBPH_aldolase subfamily using the NCBI Conserved Domains Database (https://www.ncbi.nlm.nih.gov/cdd).

References

1. González JM, Mayer F, Moran MA, Hodson RE, Whitman WB. 1997. *Microbulbifer hydrolyticus* gen. nov., sp. nov., and *Marinobacterium georgiense* gen. nov., sp. nov., two marine bacteria from a lignin-rich pulp mill waste enrichment community. Int J Syst Bacteriol 47:369–376.

2. Buchan A, Neidle EL, Moran MA. 2001. Diversity of the ring-cleaving dioxygenase gene *pcaH* in a salt marsh bacterial community. Appl Environ Microbiol 67:5801.

3. Morris JJ, Kirkegaard R, Szul MJ, Johnson ZI, Zinser ER. 2008. Facilitation of robust growth of *Prochlorococcus* colonies and dilute liquid cultures by "helper" heterotrophic bacteria. Appl Environ Microbiol 74:4530–4534.

4. Austin B, Zachary A, Colwell RR. 1978. Recognition of *Beneckea natriegens* (Payne *et al.*) Baumann *et al*. as a member of the genus *Vibrio*, as previously proposed by Webb and Payne. Int J Syst Bacteriol 28:315–317.

5. Moran MA, Buchan A, González JM, Heidelberg JF, Whitman WB, Klene RP, Henriksen JR, King GM, Belas R, Fuqua C, Brinkac L, Lewis M, Johri S, Weaver B, Pai G, Elsen JA, Rahe E, Sheldon WM, Ye W, Miller TR, Carlton J, Rasko DA, Paulsen IT, Ren Q, Daugherty SC, Deboy RT, Dodson RJ, Durkin AS, Madupu R, Nelson WC, Sullivan SA, Rosovitz MJ, Haft DH, Selengut J, Ward N. 2004. Genome sequence of *Silicibacter pomeroyi* reveals adaptations to the marine environment. Nature 432:910–913.

6. González JM, Covert JS, Whitman WB, Henriksen JR, Mayer F, Scharf B, Schmitt R, Buchan A, Fuhrman JA, Kiene RP, Moran MA. 2003. *Silicibacter pomeroyi* sp. nov. and *Roseovarius nubinhibens* sp. nov., dimethylsulfoniopropionate-demethylating bacteria from marine environments. Int J Syst Evol Microbiol 53:1261–1269.

7. Buchan A, Collier LS, Neidle EL, Moran MA. 2000. Key aromatic-ring-cleaving enzyme, protocatechuate 3,4-dioxygenase, in the ecologically important marine Roseobacter lineage. Appl Environ Microbiol 66:4662–4672.

8. Slightom RN, Buchan A. 2009. Surface colonization by marine Roseobacters: Integrating genotype and phenotype. Appl Environ Microbiol 75:6027–6037.

9. Moran MA, Belas R, Schell MA, González JM, Sun F, Sun S, Binder BJ, Edmonds J, Ye W, Orcutt B, Howard EC, Meile C, Palefsky W, Goesmann A, Ren Q, Paulsen I, Ulrich LE, Thompson LS, Saunders E, Buchan A. 2007. Ecological genomics of marine Roseobacters. Appl Environ Microbiol 73:4559–4569.

10. Schäfer H, McDonald IR, Nightingale PD, Murrell JC. 2005. Evidence for the presence of a CmuA methyltransferase pathway in novel marine methyl halide-oxidizing bacteria. Environ Microbiol 7:839–852.

11. Buchan A, Neidle EL, Moran MA. 2004. Diverse organization of genes of the β-ketoadipate pathway in members of the marine Roseobacter lineage. Appl Environ Microbiol 70:1658–1668.

12. Ankrah NYD, Lane T, Budinoff CR, Hadden MK, Buchan A. 2014. Draft genome sequence of *Sulfitobacter* sp. CB2047, a member of the Roseobacter clade of marine bacteria, isolated from an *Emiliania huxleyi* bloom. Genome Announc 2.

13. Liang C, Huang Y, Wang H. 2019. *pahE*, a functional marker gene for polycyclic aromatic hydrocarbon-degrading bacteria. Appl Environ Microbiol 85.

14. Liang C, Ye Q, Huang Y, Wang Y, Zhang Z, Wang H. 2022. Shifts of the new functional marker gene (*pahE*) of polycyclic aromatic hydrocarbons (PAHs) degrading bacterial population and its relationship with PAHs biodegradation. J Hazard Mater 437.

15. Takizawa N, Iida T, Sawada T, Yamauchi K, Wang YW, Fukuda M, Kiyohara H. 1999. Nucleotide sequences and characterization of genes encoding naphthalene upper pathway of *Pseudomonas aeruginosa* PaK1 and *Pseudomonas putida* OUS82. J Biosci Bioeng 87:721–731.

16. Kim S-J, Song J, Kweon O, Holland RD, Kim D-W, Kim J, Yu L-R, Cerniglia CE. 2012. Functional robustness of a polycyclic aromatic hydrocarbon metabolic network examined in a *nidA* aromatic ring-hydroxylating oxygenase mutant of *Mycobacterium vanbaalenii* PYR-1. Appl Environ Microbiol 78:3715.

17. Dehmel U, Engesser KH, Timmis KN, Dwyer DF. 1995. Cloning, nucleotide sequence, and expression of the gene encoding a novel dioxygenase involved in metabolism of carboxydiphenyl ethers in *Pseudomonas pseudoalcaligenes* POB310. Archives of Microbiology 1995 163:1 163:35–41.

18. Urata M, Miyakoshi M, Kai S, Maeda K, Habe H, Omori T, Yamane H, Nojiri H. 2004. Transcriptional regulation of the ant operon, encoding two-component anthranilate 1,2-dioxygenase, on the carbazole-degradative plasmid pCAR1 of *Pseudomonas resinovorans* strain CA10. J Bacteriol 186:6815–6823.

19. Kiyohara H, Torigoe S, Kaida N, Asaki T, Iida T. 1994. Cloning and characterization of a chromosomal gene cluster, *pah*, that encodes the upper pathway for phenanthrene and naphthalene utilization by *Pseudomonas putida* OUS82. J Bacteriol 176:2439–2443.

20. McKay DB, Seeger M, Zielinski M, Hofer B, Timmis KN. 1997. Heterologous expression of biphenyl dioxygenase-encoding genes from a gram-positive broad-spectrum polychlorinated biphenyl degrader and characterization of chlorobiphenyl oxidation by the gene products. J Bacteriol 179:1924–1930.

21. Fuenmayor SL, Wild M, Boyes AL, Williams PA. 1998. A gene cluster encoding steps in conversion of naphthalene to gentisate in *Pseudomonas* sp. strain U2. J Bacteriol 180:2522–2530.

22. Overhage J, Priefert H, Steinbüchel A. 1999. Biochemical and genetic analyses of ferulic acid catabolism in *Pseudomonas* sp. strain HR199. Appl Environ Microbiol 65:4837–4847.

23. Tsagogiannis E, Vandera E, Primikyri A, Asimakoula S, Tzakos AG, Gerothanassis IP, Koukkou AI. 2021. Characterization of protocatechuate 4,5-dioxygenase from *Pseudarthrobacter phenanthrenivorans* Sphe3 and *in situ* reaction monitoring in the NMR tube. Int J Mol Sci 22.

24. Neidle EL, Ornston LN. 1986. Cloning and expression of *Acinetobacter calcoaceticus* catechol 1,2-dioxygenase structural gene *catA* in *Escherichia coli*. J Bacteriol 168:815–820.

25. Le TT, Eymann C, Albrecht D, Sietmann R, Schauer F, Hecker M, Antelmann H. 2006. Differential gene expression in response to phenol and catechol reveals different metabolic activities for the degradation of aromatic compounds in *Bacillus subtilis*. Environ Microbiol 8:1408–1427.
