# Supplementary material for: Evidence for novel polycyclic aromatic hydrocarbon degradation pathways in culturable marine isolates
Source: Microbiol Spectr. 2023 Dec 12;12(1):e03409-23. doi: 10.1128/spectrum.03409-23 (PMC10783047; doi:10.1128/spectrum.03409-23)
Supplement: Table S1 — Marine strains used in this study. [file spectrum.03409-23-s0002.pdf]

Table S1: Marine strains used in this study.

**Bacteria name**	**Taxa**	**Colony Morphology**	**Genome Sequence Available?**	**16S rRNA Gene Accession Number**	**WGS Accession**	**Isolation Site**
Marinobacterium georgiense	Gamma-Proteobacteria	small, circular, translucent, white	Yes	U58339.1	NZ_CP022297.1	Coastal Georgia (USA) seawater by enrichment with lignocellulose from a pulp mill (1)
Bacillus-Clostridium strain SE165	Bacillus-Clostridium group	Large, orange colonies	No	AY038905.1	N/A	*Spartina* leaves; Skidaway Island, Georgia (USA) (2)
Bacillus-Clostridium strain SE98	Bacillus-Clostridium group	Large, white flat colonies	No	AY038926.1	N/A	*Spartina* leaves; Skidaway Island, Georgia (USA) (2)
Alteromonas macleodii strain EZ55	Gamma-Proteobacteria	Medium, white, flat colonies	Yes	EU704114	NZ_CABDXN010000001.1	Isolated from a Prochlorococcus strain MIT 9215 lab culture (3)
Vibrio natriegens ATCC 14048	Gamma-Proteobacteria	Large, beige, flat colonies	Yes	NR_117890.1	ASM145625v1	Salt marsh sediment; Sapelo Island, Georgia (USA) (4)
Rhodospirillaceae strain EZ35	Alpha-proteobacteria	Medium, beige, flat colonies	No	AF493974	N/A	Isolated from a Prochlorococcus strain MED4 lab culture (3)
Flavobacteriaceae strain EZ40	Bacteriodetes	small, flat, yellow colonies	No	EU591706	N/A	Isolated from a Prochlorococcus strain MIT 9312 lab culture (3)
Alcanivorax sp. strain EZ46	Gamma-Proteobacteria	Slow grower, small, white colonies	No	EU591711	N/A	Isolated from a Prochlorococcus strain NATL2A lab culture (3, 5)

Ruegeria pomeroyi DSS-3	Alpha-proteobacteria	Small, beige (turns dark brown), raised colonies	Yes	NR_028727	NC_003911.12	Coastal seawater; Sapelo Island, Georgia (USA), via enrichment on DMSP (6)
Citreicella sp. SE45	Alpha-proteobacteria	Medium, mucoid, whiteish-yellow colonies	Yes	AF388308	ACNW00000000.1	*Spartina alterniflora* (smooth cordgrass) detritus; southeastern US salt marshes off Skidaway Island, Georgia (USA) (2)
Sagittula stellata E-37	Alpha-proteobacteria	Small, white, raised colonies	Yes	NR_026016	NZ_AAYA00000000.1	Coastal Georgia (USA) seawater by enrichment with lignocellulose from a pulp mill (7)
Sulfitobacter sp. EE-36	Alpha-proteobacteria	Small, white colonies with orange center	Yes	AF007254	GCA_000152605.1	Salt marsh; Sapelo Island, Georgia (USA) (7)
Sulfitobacter sp. NAS-14.1	Alpha-proteobacteria	Small, white colonies	Yes	642973159 (IMG)	NZ_AALZ00000000.1	Surface waters in North Atlantic Ocean via enrichment on DMSP (8)
Ruegeria sp. TM1040	Alpha-proteobacteria	Small, yellow colonies	Yes	640715081 (IMG)	NC_008044.1	Phycosphere of the dinoflagellate *Pfiesteria piscicida* (9)
Roseovarius sp. 217	Alpha-proteobacteria	Tiny, white colonies	Yes	642973074 (IMG)	NZ_AAMV00000000.1	Surface waters near Plymouth, England enriched for growth on methyl halide (10)
Roseovarius nubinhibens ISM	Alpha-proteobacteria	Small, orange colonies	Yes	AF098495.1	GCF_000152625.1	Surface waters of the Caribbean Sea (6)

Rhodobacterales strain Y4I	Alpha-proteobacteria	Small, blue/white colonies	Yes	AF388307.1	ABXF00000000.1	Spartina alterniflora (smooth cordgrass) detritus; southeastern salt marshes off Skidaway Island, Georgia (USA) (11)
Sulfitobacter pontiacus CB-D	Alpha-proteobacteria	Small, white colonies	Yes	JN121396	IV89_000278	*Emiliania huxleyi* bloom in Raunefjorden, Norway enriched for growth on DMSP (12)
Escherichia coli DH5α	Gamma-Proteobacteria	Medium, mucoid, white colonies	Yes	KC161283	JRYM00000000.1	N/A
