# Supplementary material for: Evidence for novel polycyclic aromatic hydrocarbon degradation pathways in culturable marine isolates
Source: Microbiol Spectr. 2023 Dec 12;12(1):e03409-23. doi: 10.1128/spectrum.03409-23 (PMC10783047; doi:10.1128/spectrum.03409-23)
Supplement: Table S2 — Query sequences for protein identity searches. [file spectrum.03409-23-s0003.pdf]

Table S2: Query sequences for protein identity searches

Protein	Group	Query Strain Name	GenBank Accession Number/NCBI Reference Sequence Number	Reference
PahE-NP	PAH Hydratase Aldolase	*Novosphingobium pentaromativorans* US6-1	AIT82654.1	Liang et al., 2019; Liang et al., 2022 (13, 14)
PahE-RO	PAH Hydratase Aldolase	*Rhodococcus opacus* B4	BAH47216	Liang et al., 2019; Liang et al., 2022 (13, 14)
PahE-MV	PAH Hydratase Aldolase	*Mycobacterium vanbaalenii* PYR-1	ABM11319	Liang et al., 2019; Liang et al., 2022 (13, 14)
PahE-PA	PAH Hydratase Aldolase	*Pseudomonas aeruginosa* PaK1	BAA12246.1	Takizawa et al., 1999 (15)
NidA	Gram + PAH/Phthalate	*Mycobacterium vanbaalenii* PYR-1	AF249301.2	Kim et al., 2012 (16)
PobA	I	*Pseudomonas pseudoalcaligenes* POB310	CAA55400.1	Dehmel et al., 1995 (17)
AntA	II	*Pseudomonas resinovorans* CA10	WP_011077861.1	Urata et al., 2004 (18)
PahAc	III	Pseudomonas putida OUS82	BAA20391.1	Kiyohara et al., 1994 (19)
BphA1	IV	*Rhodococcus globerulus* P6	CAA56346.1	McKay et al., 1997 (20)
NagG	Salicylate	*Ralstonia* sp. U2	AAD12607.1	Fuenmayor et al., 1995 (21)
PcaH	Protocatechuate 3,4-Dioxygenase	*Pseudomonas* sp. Strain HR199	CAB43484.1	Overhage et al., 1999 (22)
PcaA/LigB	Protocatechuate 4,5-Dioxygenase	*Pseudarthrobacter phenanthrenivorans* Sphe3	ADX75303.1	Tsagogiannis et al., 2021 (23)
CatA	Catechol 1,2-Dioxygenase	*Acinetobacter calcoaceticus*	SUU57696.1	Neidle &Ornston, 1986 (24)
CatE/YfiE	Catechol 2,3-Dioxygenase	*Bacillus subtilis* 168	BAA09109.1	Thi Tam et al., 2006 (25)
